# Extreme heat preparedness and response implementation: a qualitative study of barriers, facilitators, and needs among local health jurisdictions in the United States

**DOI:** 10.1088/2752-5309/adf08b

**Published:** 2025-07-23

**Authors:** Jessica C Kelley, Cat Hartwell, Evan C Mix, Chelsea Gridley-Smith, Gregory A Wellenius, Amruta Nori-Sarma, Jeremy J Hess, Nicole A Errett

**Affiliations:** 1 Department of Environmental and Occupational Health Sciences, University of Washington School of Public Health, Seattle, WA, United States of America; 2 National Association of County and City Health Officials, Washington, DC, United States of America; 3 Center for Climate and Health, Boston, MA, United States of America; 4 Department of Environmental Health, Boston, MA, United States of America; 5 Center for Climate, Boston, MA, United States of America; 6 Department of Global Health, University of Washington School of Public Health, Seattle, WA, United States of America; 7 Department of Emergency Medicine, University of Washington School of Medicine, Seattle, WA, United States of America

**Keywords:** extreme heat events, local health jurisdictions, public health, public health emergency preparedness

## Abstract

Extreme heat events (EHEs) are the deadliest weather hazards in the United States (U.S.). Local health jurisdictions (LHJs) in the U.S. are frontline responders during EHEs and other public health emergencies. This study aims to clarify the factors influencing EHE preparedness and response implementation. From January to March 2023, we conducted and thematically analyzed four focus group discussions with 17 representatives from U.S. LHJs. The Consolidated Framework for Implementation Research was used to guide the discussion. Participants described barriers, facilitators, and needs surrounding extreme heat preparedness and response implementation. The focus group discussions identified four factors that influence EHE preparedness and response implementation: local conditions (environmental, political, planning); engaging communities and tailoring strategies; partnerships and relational connections; and available resources. Focus group discussions emphasized the need for EHE preparedness and response activities to be targeted and scaled to the unique climate, population, and needs of the implementing jurisdiction. Local conditions, community engagement, partnerships, and available resources shape LHJ priorities. The study emphasizes the need for scalable resources and comprehensive plans, and identifies research gaps to be addressed in the future.

**Abbreviations**
CDC: Centers for Disease Control and Prevention
CFIR: Consolidated Framework for Implementation Research
EHE: Extreme heat event
HAP: Heat action plan
LHJ: Local health jurisdiction
NACCHO: National Association of County and City Health Officials
NOAA: National Oceanic and Atmospheric Administration
NWS: National Weather Service

## 1. Introduction

Average global temperatures are rising due to anthropogenic climate change and each of the last four decades has been warmer than any prior decade since 1850 [1]. The frequency and severity of EHEs is increasing, putting more people at risk of dangerous and even fatal heat exposure [1–3]. Indeed, extreme heat is the deadliest weather event in the United States (U.S.) and is associated with excess hospital admissions and emergency department visits [4, 5]. Even more concerning, global CO2 emissions are increasing, suggesting that warming will continue to accelerate along with harm to human health [6].

Extreme heat poses significant health risks to various at-risk populations and challenges to critical infrastructure [7]. Exposure to extreme heat causes conditions such as heat exhaustion, heatstroke, dehydration, and renal disease [5, 8, 9]. Certain populations are at greater risk than others including older adults, children, individuals with disabilities, individuals with chronic health conditions, persons experiencing homelessness, members of racial and ethnic minority groups, low-income individuals, socially isolated individuals, and outdoor workers [8, 10–12]. Extreme heat also strains or overwhelms critical built infrastructure that is not engineered to withstand high temperatures [13] [14], and can disrupt the ability of healthcare facilities to provide routine services [2].

In the U.S., LHJs are critical first responders during disasters and emergencies. The approximately 2,800 LHJs across the U.S. coordinate and implement public health interventions to prevent adverse health consequences of EHEs and other emergencies [15–17]. This requires collaboration with cross-sector partners (e.g. public health and emergency management) at all levels of government, often based on plans and policies developed in advance. In this way LHJs seek to equip communities with the resources, strategies, and protocols needed to respond effectively in times of crisis [18]. During emergencies, LHJs lead efforts to provide timely and accurate information, implement preventive measures, coordinate medical services, and support at-risk populations [19].

While jurisdictions across the U.S. are concerned about EHEs, many have been slow to implement heat preparedness and response strategies [15, 20]. The World Health Organization has issued guidance regarding public health response to EHEs that identifies the core elements of effective HAPs [21]. However, a 2011 survey of 190 local public health and emergency response agencies examining county-level heat preparedness and responses found that only 40% had formal HAPs; more populous counties were more likely to have formal plans in place [20]. The same study found that only 24% of jurisdictions with HAPs actually activated their plans during a summer of record heat [20]. HAPs may be growing more common: in a 2021 study, 60.5% of 38 surveyed jurisdictions with populations of at least 200 000 reported having written HAPs [22]. However, the activities included in these HAPs (e.g. heat vulnerability mapping, cooling centers, surveillance, evaluation) and the degree to which individual jurisdictions pursued these activities varied greatly [22].

LHJs are ideally positioned to address the health consequences of EHEs because they know the communities they serve [19]. Some LHJs are already acting on their HAPs by establishing formal definitions of extreme heat, conducting surveillance, developing plans to open cooling centers, facilitating well-being checks, and conducting evaluations [23]. While prior research has identified EHE strategies and activities LHJs pursue, it is not clear what barriers and facilitators influence LHJs’ implementation of EHE preparedness and response [22]. Accordingly, this study aims to identify the factors that affect LHJ implementation of EHE preparedness and response strategies. Doing so is important because while climate change is occurring globally, the resulting health impacts are experienced locally; LHJs bear the brunt of the responsibility for educating the public on the nature of these local impacts, preparing for them, and marshaling a response, so they must be provided with the tools necessary to do so [24–27].

## 2. Methods

We conducted focus group discussions among local public health officials in the U.S. to explore factors that influenced agency-level engagement in EHE preparedness and response. On 6 January 2023, the University of Washington Human Subjects Division determined this study to be human subjects research that qualifies for exempt status (STUDY00016892).

### 2.1. Data collection

We used a purposive sampling strategy facilitated by research partners at the NACCHO, a professional advocacy organization that represents over 3300 local health departments across the U.S [28]. LHJ representatives were recruited from several NACCHO workgroups: preparedness planning, outcomes, and measurement; preparedness policy advisory; environmental public health; and global climate change. We did not require participants to be engaged directly in EHE preparedness and response, but recruitment targeted workgroups focused on preparedness, environmental health, and climate change. Representatives of state and federal agencies were excluded. We invited members of these workgroups to participate by email.

We held four focus group discussions on Zoom between January 23 and 10 February 2023. Each lasted between 40 and 60 min. Researchers provided background information on the study and intended uses of the data collected (Supplemental Material 1). Participants were able to ask questions before providing verbal consent to participate and agreeing to be audio recorded.

This qualitative study was informed by implementation science, which examines approaches to improve the uptake of research findings in practice [29]. Focus group discussions were guided by version 2.0 of the CFIR, which is the most current version of the CFIR that reflects recent revisions to incorporate user feedback [30]. It uses five domains to characterize and understand barriers to and facilitators of implementation: ‘Innovation,’ or the thing being implemented; ‘Inner Setting,’ or the environment in which the innovation is being implemented; ‘Outer Setting,’ or the context in which the inner setting takes place; ‘Individuals,’ or the characteristics of the people implementing the innovation; and ‘Implementation Process,’ or the strategies and actions those people take to implement the innovation [30]. We did not investigate the ‘Innovation’ domain (i.e. the thing being implemented) because no single innovation was under consideration; rather, the study considered determinants of the implementation of a variety of activities constituting EHE preparedness and response more broadly (Supplemental Material 1).

### 2.2. Data analysis

We recorded audio of all focus group discussions for professional transcription. A codebook was developed deductively based on the CFIR 2.0 framework (supplemental material 2) [30]. Cleaned transcripts were imported into NVivo [31], Two researchers (JK and CH) used NVivo to code the transcripts on a consensus basis.

Researchers applied the framework method for qualitative research, which uses a matrix to organize data and compare and contrast across units of analysis [32]. The matrix was organized with codes categorized in rows, focus groups in columns, and focus-group-level code summaries in corresponding cells. Data extracted and analyzed via the matrix were synthesized into a narrative analytic memo, highlighting commonalities and counterpoints. Researcher JK led the analysis, and CH compared summaries against raw data and the analytic memo against the summaries to ensure consistency.

## 3. Results

**Figure 1.**
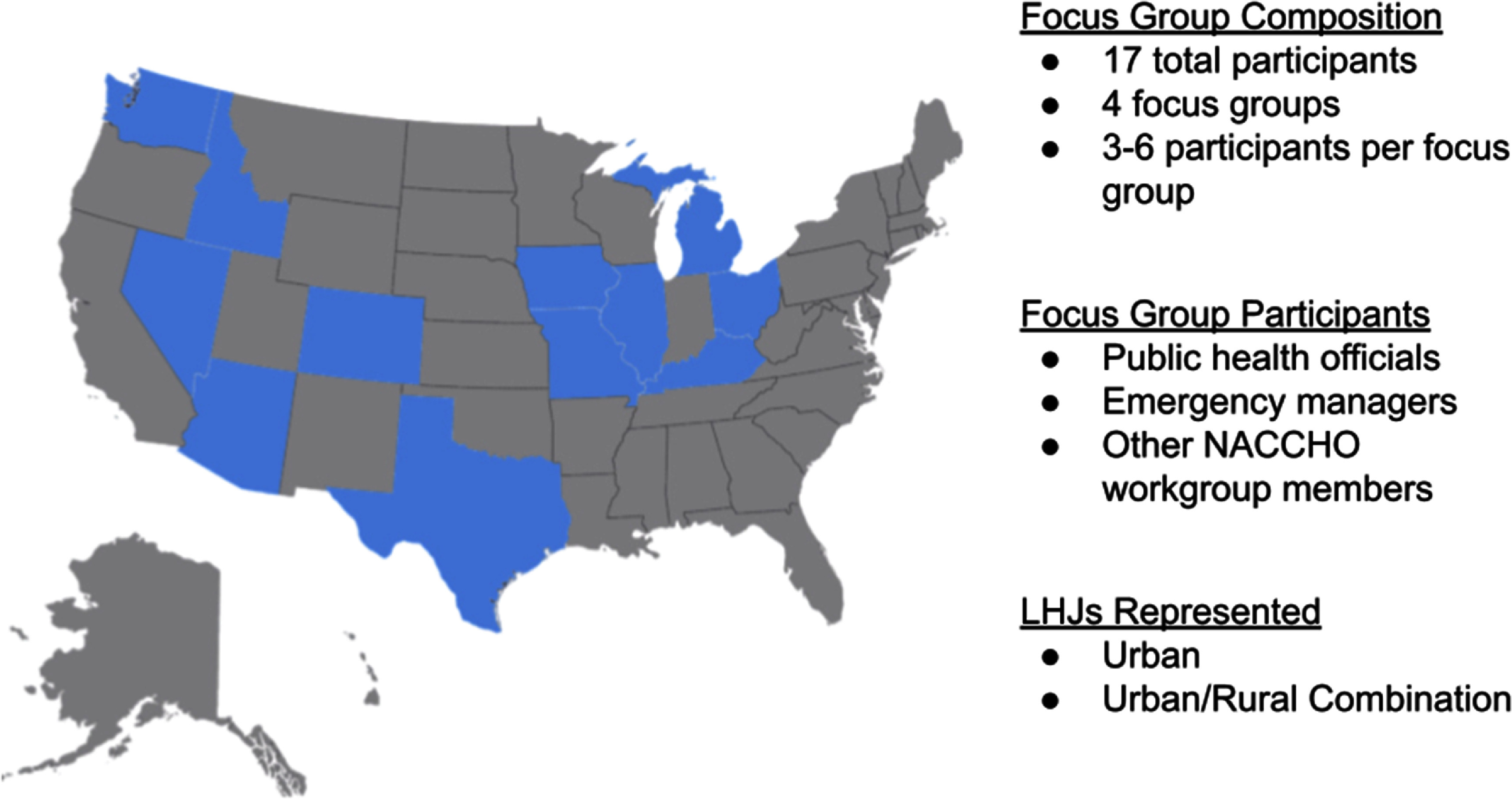
Focus group participation.

The study team conducted four focus group discussions with a total of 17 participants in January and February 2023 (figure 1). Each focus group comprised three to six participants, including public health officials, emergency managers, and other contributors actively involved in NACCHO workgroups. Participants represented cities, counties, and districts (e.g. LHJs serving multiple counties within a region) across 12 U.S. states, providing insights from various climatically and demographically distinct regions and service areas. Focus groups included representatives of LHJs serving either urban residents or a combination of rural and urban residents.

Table 1 summarizes the barriers to and facilitators of EHE preparedness and response that emerged from these discussions. All information presented here originates from specific comments and recommendations by focus group members.

**Table 1.** Barriers and facilitators.

Themes	Barriers	Facilitators
Local conditions: environmental	> Varied risk perception across regions > EHE is an established concern in hotter regions and not always considered an emergency alone > Delayed benefits and disproportionate focus on long-term (e.g. tree planting) as opposed to short- and medium-term strategies	> Increased awareness of regional heat impacts and recognition of consistent heat challenges > Addressing intersections with other emergencies and compound hazards > Recognition of unique challenges and adaptation

Local conditions: political	> Local heat indices with temperature thresholds that do not activate early enough to mitigate heat-health impacts > Variable to minimum support from decision-makers to prioritize EHEs > Political climates affect engagement with EHE strategies > Balancing economic drivers with public health priorities (e.g. canceling major outdoor events like sports or concerts)	> Establishing clear definitions and action thresholds > Consideration of lower thresholds for at-risk groups > Support from local leadership and elected officials > Collaboration with utility companies for coordinated action

Local conditions: planning	> Lack of data and limited heat-specific surveillance > Insufficient attention to specific at-risk populations during EHEs > Limited knowledge and information for effective decision-making > Lack of coordination and resources for local planning efforts	> Access to resources, tools, and collaboration for surveillance > Recognition and focus on at-risk populations in planning > Syndromic surveillance to assess and address health impacts > Grant funding to support climate resilience, community engagement, and professional development > Consideration of environmental factors in local planning efforts > Training on extreme heat health effects and strategies > Incorporation of public health in regional planning

Engaging & tailoring strategies	> Stigma and accessibility issues for individuals experiencing homelessness > Lack of effective communication among public agencies > Absence of culturally relevant messaging and translations > Jurisdictional gaps in addressing needs of vulnerable groups	> Designated access times to cooling centers during EHEs for at-risk populations (i.e. individuals experiencing homelessness) > Improvement in coordination through effective communication strategies > Inclusion of culturally relevant messaging and translations in planning > Collaborative efforts of governmental and non-governmental organizations > Building community support for heat initiatives > Effective public education and risk communication
Partnerships & relational connections	> Discontinuation of specialized teams and outreach capabilities without COVID-19 funding > Limited partnerships between public health and city planning > Challenges in connecting with LHJs experienced in responding to EHEs	> Continued funding and support for specialized teams > Promotion of partnerships between public health and city planning > Facilitation of connections with LHJs for resource and knowledge sharing > Emphasis on both formal and informal cooling centers > Engagement of hospitals and healthcare systems in surveillance efforts > Recognition of elected officials as critical to implementation efforts > Collaboration with power providers for messaging and response

Available resources	> Variations in resource availability and gaps in heat preparedness > Public health workforce crisis constraining capacity and involvement > Prioritization of easier projects over strategic efforts > Limited cooling spaces for at-risk populations and weekend limitation with business closures and reduced staffing > Limited availability of resources for translation and tailoring messaging	> Strategies to ensure support for at-risk groups during EHEs > Seeking small grants for professional development and community engagement from organizations like NOAA, EPA, and CDC > Flexible local planning to address diverse preferences and circumstances > Acknowledgment of political and legislative factors limiting funding > Considering additional strategies for cooling center availability during weekends and other times when businesses are closed or when staffing may be reduced

### 3.1. Local conditions: environmental

All focus group participants expressed concerns about the current and near-future impacts of EHEs. Participants from historically temperate regions such as the Pacific Northwest reported extreme heat as an emerging issue. This region has experienced more days of extreme heat in recent years. On the other hand, for participants from the South and Southwest, heat is an established concern. Participants discussed how the prevalence of air conditioning and acclimatization to hotter temperatures lead residents of those regions to consider heat-related illness less risky, even though not all residents have access to air conditioning.

Heat becomes even more challenging when combined with other emergencies and hazards. For example, two focus groups highlighted the impact of wildfires and rising ozone levels on air quality, particularly when temperatures do not drop overnight—residents are less likely to open windows for heat relief during periods of bad air quality, making EHEs that coincide with air-quality events more problematic. Participants from two focus groups reported that in hotter regions, an EHE is not considered an emergency unless it coincides with large-scale power outages preventing the use of air conditioning as a cooling intervention.

All four focus groups discussed the significance of regional climate differences and differences between rural and urban communities. Participants stressed the importance of climate adaptation and mitigation strategies that consume minimal water, particularly in arid regions. These regions require drought-resistant landscaping (e.g. plants that need less water), which may not provide sufficient cooling relief. In urban areas, heat islands can significantly raise temperatures. While tree planting is a popular strategy to reduce the urban heat island effect, participants in two focus groups noted that it may not yield immediate or near-term benefits. In rural agricultural areas in the Midwest, the evaporation of water from vast quantities of maturing corn, known as ‘corn sweat,’ increases humidity from mid-July through August.

### 3.2. Local conditions: political

Focus group participants described significant variation between LHJs concerning governance, roles, and responsibilities related to extreme heat preparedness and response at the county, city, and state levels. Participants from one focus group noted that responsibility for EHE activities often rests with emergency management agencies, with limited involvement from public health departments.

All four focus groups discussed the political conditions necessary to support EHE preparedness and response. The extent to which an LHJ is able to engage with climate change and adopt extreme heat mitigation strategies is influenced by the local political climate, sentiments of elected officials, and LHJ leadership. For example, lowering the temperature threshold at which response measures can be activated may require buy-in from elected officials who have engaged little with the topic and face many competing priorities. Participants in three of four focus groups mentioned that decision-makers (i.e. elected officials and LHJ leaders) who are not engaged can impede preparedness and response implementation. Conversely, participants in two focus groups explained how receiving support from local decision-makers to plan and implement mitigation strategies or lower heat-action thresholds led to more resources (e.g. funding, staffing, and other human resources), opening possibilities like providing free public transportation during EHEs.

Participants also discussed how politics influence the implementation of specific risk reduction strategies. For example, participants in one focus group described the difficulty of canceling outdoor events like a major concert or sports event due to extreme heat. Decisions like this are not based solely on temperature thresholds or a public health official’s order—there are also political and economic considerations.

### 3.3. Local conditions: planning

All focus groups discussed the importance of targeted planning that addresses the unique needs of specific populations, enhances the built environment, and proactively mitigates public health risks during EHEs. Participants in two focus groups revealed that their jurisdictions had standalone plans specifically tailored to extreme heat. However, the majority of participants in all focus groups reported that their jurisdictions lacked such plans, instead considering extreme heat as part of their all-hazard preparedness plans which often includes extreme cold as well.

According to participants, it is essential to define extreme heat precisely in order to determine when response is appropriate. Participants from three focus groups use the NWS ‘heat index’ to assess heat risk; others utilize locally defined thresholds. However, participants from the South and Southwest reported that heat-related health risks arise in their communities even when temperatures are below these thresholds, especially for at-risk groups. One LHJ developed a heat vulnerability index map to identify groups that are more susceptible to extreme heat.

Participants emphasized the value of syndromic surveillance to assess the impacts of heat on public health. Three focus groups discussed collaborating with hospitals to collect heat-specific syndromic data; two discussed leveraging syndromic heat data when engaging elected officials; and one discussed using these data to create a heat vulnerability index map to calibrate heat action thresholds. Planning strategies should include resources and tools to engage communities, heat-specific syndromic surveillance, region-specific guidance on prevalence of extreme heat, climate epidemiology, collaboration across health systems, and access to information necessary to influence decision-makers. Other components of local planning include the availability and coordination of cooling centers and monitoring drastic temperature changes for effective messaging and risk communication.

Finally, participants identified important planning facilitators. Grant funding facilitates effective planning and supports endeavors to foster climate resilience, conduct community environmental and health needs assessments, administer surveys, and pursue professional development. The presence of syndromic surveillance, cooling center availability, messaging, and shelter placement were also identified as essential elements of any successful heat preparedness and response implementation.

### 3.4. Engaging communities & tailoring strategies

Participants from all four focus groups identified the importance of engaging community members and tailoring strategies to meet the needs of individuals and groups, including migrants, tourists, non-English speakers, refugees, persons experiencing homelessness, and outdoor workers. Participants in one focus group identified specific considerations for areas with transient populations due to tourism and visitation, including the need for safety measures during outdoor activities–especially those involving high alcohol intake. Individuals experiencing homelessness are sometimes barred from accessing shelters and cooling centers due to behavioral issues and stigma; participants in one focus group suggested allowing access at designated times during periods of high temperature. Finally, participants highlighted the need for messaging that is culturally relevant to local demographics and materials translated to languages other than English.

### 3.5. Partnerships & relational connections

Participants across all focus groups wanted to partner with counterparts in other jurisdictions, agency and elected officials, and business and community partners to improve EHE response. Participants overwhelmingly expressed interest in connecting with LHJs that have experience responding to EHEs to share lessons learned. Elected officials were identified as critical to short- and long-term implementation efforts. Participants from one focus group also expressed the need for additional government partnerships, particularly between public health and city planning, to address social determinants of health and incorporate local climate epidemiology and heat-related syndromic surveillance into preparedness efforts. Finally, participants noted the importance of engaging hospitals and healthcare systems, which play a role in surveillance and health impact evaluation.

In the area of heat communication, participants discussed partnerships with entities such as the NWS, emergency management, nongovernmental organizations, and elected officials. They also acknowledged the importance of messaging partners, including local news media and meteorologists, for disseminating heat information. Participants identified the NWS as a particularly valuable resource for monitoring extreme weather and because of its heat index.

All focus groups discussed implementing both formal and informal cooling centers. Formal cooling centers are supported by the LHJs, often in collaboration with community partners such as libraries, schools, faith-based organizations, and community organizations. Informal cooling centers such as shopping malls and movie theaters are not activated as a part of the LHJ heat response efforts, but are often preferred because they are convenient spaces with which people are already familiar.

Finally, participants in all four focus groups emphasized the importance of coordinating with power and other utility companies. Planned and unplanned outages have compounding effects during EHEs. Power providers are important to messaging, anticipating, and responding to these events; arranging energy sourcing and financing for utility assistance; and planning for capacity constraints due to increasing consumption.

### 3.6. Available resources

Participants pointed out that individual LHJs have different levels of access to resources and services in heat preparedness and response implementation. However, certain resources are consistently less accessible. For example, many LHJs lack the funding and capacity to translate messaging and tailor outreach to different communities and at-risk groups, implement heat-specific syndromic surveillance and community assessments and support heat action more broadly. Participants reported a pressing need for resources and services to ensure that at-risk groups receive necessary support during EHEs. Two focus groups described specialized teams established to engage with the community that were supported by COVID-19-specific funding; these teams could be discontinued along with this funding.

Participants in one focus group discussed the ongoing public health workforce crisis, which is straining the bandwidth of staff members and impeding their involvement in heat preparedness and response initiatives. Participants in another focus group noted that projects unrelated to extreme heat are often prioritized over strategic efforts to enhance EHE preparedness because they are easier to complete or require fewer resources. Participants also acknowledged that political and legislative factors could limit funding for LHJ climate strategies. Participants recommended applying for small grants, such as those provided by the NOAA, Environmental Protection Agency, and CDC to support professional development, community engagement, community assessments, and surveillance–a strategy that prior research has commended [26] for its potential to help LHJs prepare for climate-related hazards like extreme heat.

The limited availability of cooled physical spaces to shelter people who lack access to air conditioning emerged as a significant constraint on the implementation of EHE preparedness and response. Participants in all focus groups discussed difficulties accessing adequate shelter capacity to meet the needs of unhoused populations during EHEs. Additionally, space is more limited on weekends than on weekdays due to business closures and reduced staffing, including the LHJ staff who plan and implement response measures.

## 4. Discussion

This study leveraged the CFIR 2.0 framework to identify factors that impede or facilitate the implementation of extreme heat preparedness and response activities by LHJs. We find that the degree to which LHJs are able to implement extreme heat preparedness and response activities is multifactorial, and the determinants of this success are both complex and highly localized. On the whole, our findings suggest that LHJs are generally aware of the increasing threat EHEs pose to public health and understand how different preparedness and response activities could mitigate that threat, but sometimes struggle to implement measures to mitigate that threat. Here we identify the most important barriers to EHE response implementation reported by our participants and suggest some strategies to overcome them.

Many of the barriers our participants identified resolve to a lack of resources. Participants in our study are aware of the threat posed by EHEs, but other, more immediate threats may take precedence over planning for heat when funding and staff capacity are limited. One of the most important ways to ease the implementation of extreme heat preparedness and response, then, is to make more funding and other resources available to support this work. Some jurisdictions have explored the use of grants specifically earmarked to increase LHJ capacity to address the health impacts of climate change. A 2019 study identified six states that used funding from CDC’s Building Resilience Against Climate Effects (BRACEs) framework to make small grants to LHJs to implement a variety of climate-related capacity-building, partnership development, and planning activities; recipients of this funding largely considered it effective in supporting the implementation of climate mitigation and adaptation strategies [26]. A similar approach could benefit LHJs struggling to find the funding and capacity to prioritize EHE-related implementation activities. When state and federal authorities earmark funding for these specific actions, issues concerning resource allocation either by LHJs themselves or by other public officials are mitigated. Such funding would ideally be ongoing or renewable to ensure program sustainability and sufficient in amount to enable the hiring of staff dedicated to implementation.

Devoting sufficient resources to LHJs would pave the way for other policy changes to improve EHE preparedness and response implementation. For example, participants indicated that primary responsibility for EHE preparation and response often falls to emergency managers rather than health officials. Better-resourced LHJs would be positioned to assume more responsibility for EHE preparation and response from those officials, increasing attention to the public health impacts of these events. Ideally, these efforts would be pursued in partnership between local public health and emergency management officials.

Partnership was a theme of our findings more broadly. Participants indicated that LHJs rely, when implementing EHE mitigation and response measures, on relationships with other local governmental actors (e.g. emergency management and urban planning) and elected officials, but also on those with public utilities, private businesses, and community and religious groups. All of these entities have vested interests in effective EHE response, and each brings a different combination of expertise and resources to the table. These relationships could be formalized through the creation of standing committees, task forces, or other ongoing partnership structures dedicated to implementing EHE preparedness and response.

Other implementation barriers our participants identified could be addressed by making better information available to the right decision-makers. While participants in our study generally understood both the health impacts of EHEs and how to address them, the same may not be true of other local officials who can influence implementation activities. LHJ officials typically need buy-in from the elected officials to whom they report in order pursue the implementation activities described here, which can be difficult when those officials have competing interests and are not aware of the EHE threat to public health. Equipping LHJs with information intended for this audience and making that information otherwise available to local elected officials may help to remove this barrier to action.

New resources are becoming available to support those engaged in EHE preparedness and response. In 2022, the Biden Administration launched HEAT.gov–an all-purpose extreme heat resource that includes information about EHEs, including vulnerable populations, heat islanding, and a variety of planning tools [33]. NACCHO’s Toolbox, a collection of tools created by and for public health agencies, is another example of an important resource for information of this kind [34]. However, these compendiums mostly provide resources designed for practitioners rather than elected officials. Sample policy briefs, presentation templates, and other resources targeting this audience could help educate key decision-makers on the growing EHE threat, building political will to support more robust responses.

Our study supports findings from prior research about the important role that LHJs play in EHE preparedness and response and the need to tailor strategies to local conditions in order to fill preparedness and response implementation gaps and support evaluation of EHE-related activities. Focus group participants called for standardized approaches to heat planning that can be adapted to meet specific community and at-risk group needs. Prior research has demonstrated the importance of community engagement in mitigating the impacts of extreme heat [35, 36]. For example, the Nature’s Cooling Systems project in Phoenix, Arizona, used workshops and meetings in three heat-vulnerable neighborhoods to co-produce hyper-local HAPs designed by and for these communities [35].

Future studies should investigate how community-based organizations, academic institutions, and government agencies can support extreme heat initiatives. Understanding the nuances of partnerships and collaborations can illuminate the pathways to increased LHJ capacity and inform the development of sustainable and effective multi-sector approaches to heat preparedness and response and other climate-related public health interventions. Moreover, professional associations (e.g. NACCHO) and government agencies (e.g. CDC) should consider developing tools to help LHJs integrate evidence-based approaches to heat preparedness and response into their extreme heat planning [23].

### 4.1. Limitations

The strengths of qualitative research include the ability to deeply explore contextual issues driving a particular phenomenon. Non-random, purposive sampling recruits participants with unique perspectives or lived experience to provide such insights; however, for the same reasons, resulting information may not be generalizable. While we attempted to ensure that focus group participants came from diverse geographic regions, LHJs of different sizes, and LHJs at distinct stages of EHE preparedness and response, the small sample size in this study may limit the representativeness of our data. Participants were self-selected from specific workgroups at a single organization, NACCHO, which could further limit generalizability. Finally, the study’s timeframe was limited to January and February 2023. During this period, the attention of LHJs may have been directed towards more seasonally relevant hazards, such as cold winter temperatures, which may have discouraged some LHJs from participating or affected the participants’ ability to recall specific details about their jurisdictions’ EHE preparedness and response.

## 5. Conclusion

Leveraging an implementation science framework, this qualitative research identifies factors influencing EHE preparedness and response implementation from the LHJ perspective. Local conditions (environmental, political, planning); community engagement and tailored approaches; partnerships and connections; and available resources were all reported to influence LHJ prioritization and/or implementation of EHE preparedness and response activities. Participants suggested the need for greater access to data on local health risks from heat, resources and tools to engage partners, collaboration across health systems, knowledge sharing, and other resources to support implementation and evaluation of EHE preparedness and response activities. Our findings suggest that promoting facilitators of EHE preparedness and response implementation efforts will require scalable resources and the development of comprehensive plans that safeguard at-risk populations. These findings can inform policy makers, public health practitioners, emergency management professionals, and other partners seeking to strengthen LHJs’ capacity to mitigate the health impacts of EHEs. Research gaps that could be addressed in future studies include evaluating characteristics of EHE preparedness and response innovations and applied research and evaluative efforts that are scalable to support state, local, tribal, and territorial health jurisdictions to respond to EHE health threats.

## Data Availability

The data cannot be made publicly available upon publication because they contain sensitive personal information. The data that support the findings of this study are available upon reasonable request from the authors.
